# Association of retinal nerve layers thickness and brain imaging in healthy young subjects from the i‐Share‐Bordeaux study

**DOI:** 10.1002/hbm.26412

**Published:** 2023-07-04

**Authors:** Sara Cristina Lima Rebouças, Fabrice Crivello, Ami Tsuchida, Christophe Tzourio, Cédric Schweitzer, Jean‐François Korobelnik, Cécile Delcourt, Catherine Helmer

**Affiliations:** ^1^ University of Bordeaux, INSERM, BPH, U1219 Bordeaux France; ^2^ University of Bordeaux, CNRS, CEA, IMN, GIN Bordeaux France; ^3^ Bordeaux University Hospital Bordeaux France; ^4^ Department of Ophthalmology Bordeaux University Hospital Bordeaux France

**Keywords:** brain, brain imaging, magnetic resonance imaging, optical coherence tomography, retinal layers, young

## Abstract

Given the anatomical and functional similarities between the retina and the brain, the retina could be a “window” for viewing brain structures. We investigated the association between retinal nerve fiber layers (peripapillary retinal nerve fiber layer, ppRNFL; macular ganglion cell‐inner plexiform layer, GC‐IPL; and macular ganglion cell complex, GCC), and brain magnetic resonance imaging (MRI) parameters in young health adults. We included 857 students (mean age: 23.3 years, 71.3% women) from the i‐Share study. We used multivariate linear models to study the cross‐sectional association of each retinal nerve layer thickness assessed by spectral‐domain optical coherence tomography (SD‐OCT) with structural (volumes and cortical thickness), and microstructural brain markers, assessed on MRI globally and regionally. Microstructural MRI parameters included diffusion tensor imaging (DTI) and Neurite Orientation Dispersion and Density Imaging (NODDI). On global brain analysis, thicker ppRNFL, GC‐IPL and GCC were all significantly associated with patterns of diffusion metrics consistent with higher WM microstructural integrity. In regional analyses, after multiple testing corrections, our results suggested significant associations of some retinal nerve layers with brain regional gray matter occipital volumes and with diffusion MRI parameters in a region involved in the visual pathway and in regions containing associative tracts. No associations were found with global volumes or with global or regional cortical thicknesses. Results of this study suggest that some retinal nerve layers may reflect brain structures. Further studies are needed to confirm these results in young subjects.

## Graphical abstract

In a sample composed exclusively of young healthy subjects, we found significant associations between retinal nerve layers with occipital brain volumes, and with brain diffusion MRI parameters, globally and in regions containing associative, commissural tracts, and in a projection area involved in the visual pathway. Our results suggest common maturational processes in the retina and the brain. Further studies are needed to confirm these results and to study common factors that might explain the parallel maturation of the retina and the brain.

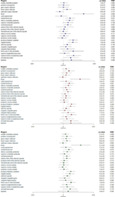

## 1 INTRODUCTION

The eye and the central nervous system (CNS) are anatomically connected because of their common embryological origin. The retina is known to be an extension of the brain and could act as a “window” for assess brain structures (London et al., 2013). The accessibility of retinal components and the possibility of an effective evaluation of retinal structures through advanced ophthalmic imaging techniques, including retinal layer visualization, may provide a useful tool to serve as a model of neurodegenerative processes (Cheung et al., 2017; Snyder et al., 2021). Optical coherence tomography (OCT) is a noninvasive imaging technique extensively applied in ophthalmology for retinal layer examination, in particular spectral‐domain optical coherence tomography (SD‐OCT). This technique provides high resolution images and quantitative assessment of the retina layers on the micron scale (Fujimoto, 2003; Huang et al., 1991).

Retinal layers gradually thin with age (Cheung et al., 2011; Duan et al., 2010; Hoffmann et al., 2018; Mauschitz et al., 2018; Nousome et al., 2021; von Hanno et al., 2017). Previous studies have evaluated retina–brain connections, analyzing retinal layers thickness, assessed by OCT, in relation to brain markers obtained with magnetic resonance imaging (MRI) (Barrett‐Young et al., 2023; Casaletto et al., 2017; Chua et al., 2021; Jorge et al., 2020; Mauschitz et al., 2022; Mejia‐Vergara et al., 2021; Méndez‐Gómez et al., 2018; Mutlu et al., 2017, 2018; Ong et al., 2015; Shi et al., 2020). Retinal layers thinning were associated with brain volume reduction or cortical thinning (Barrett‐Young et al., 2023; Casaletto et al., 2017; Chua et al., 2021; Jorge et al., 2020; Mauschitz et al., 2022; Mejia‐Vergara et al., 2021; Méndez‐Gómez et al., 2018; Mutlu et al., 2017, 2018; Ong et al., 2015; Shi et al., 2020), and with white matter (WM) microstructure alterations (Mauschitz et al., 2022; Méndez‐Gómez et al., 2018; Mutlu et al., 2017, 2018). However, prior studies were mainly carried out in persons with neurological conditions, or middle‐aged/older adults.

Very few studies have examined retina‐brain links among healthy young adults. Those few with young participants had small sample sizes in the young age‐range (less than 20) as they also included middle aged and older people (Jorge et al., 2020; Mejia‐Vergara et al., 2021). They reported correlations between retinal layers thickness and cortical thickness in the primary visual cortex (Jorge et al., 2020) or grey matter volumes in different brain regions (Mejia‐Vergara et al., 2021). A better understanding of these relationships in young adults would make it possible to determine whether the associations between retinal and cerebral structures are driven by aging or specific neuropathological processes, or also exist early in life.

In this paper, we aimed to study the association of retinal nerve layers thickness, assessed by OCT, with structural and microstructural brain MRI markers in healthy young adults. We hypothesized that individual differences in the retinal structural characteristics might closely reflect those at the brain level, given their common embryological origin. The cerebral cortex and the white matter change throughout the lifespan. Previous studies reported age‐related patterns of macrostructural and microstructural changes in young adults, including cortical thinning, reduction of grey matter volumes, increase in white matter volumes and changes in microstructural properties of white matter (Coupé et al., 2017; Kochunov et al., 2011; Koolschijn & Crone, 2013; Lebel et al., 2012; Lebel & Beaulieu, 2011; Pines et al., 2020; Simmonds et al., 2014; Tamnes et al., 2010; Westlye et al., 2010; Yeatman et al., 2014; Zhou et al., 2015).

We aimed to investigate whether these changes could be associated with retinal layer thickness. In line with previous studies (Casaletto et al., 2017; Mutlu et al., 2017, 2018; Ong et al., 2015), we hypothesized that retinal macular layers (ganglion cell‐inner plexiform (GC‐ILP) and ganglion cell complex (GCC)), mostly composed of cell bodies of retinal ganglion cells neurons reflect more gray matter (GM) volume and cortical thickness, while the peripapillary retinal nerve fiber layer (ppRNFL), composed primarily of retinal ganglion cell axons may reflect more the volume or microstructural properties of the WM. Thanks to the i‐Share cohort, we had the opportunity to analyze a large population of young adults, with accurate measures of retinal layers and brain imaging, including novel diffusion‐based measures to characterize the WM microstructure.

## 2 MATERIALS AND METHODS

### 2.1 Study population

The study population consisted of students from the i‐Share cohort project (Internet‐based Students HeAlth Research Enterprise, www.i-share.fr), which is an ongoing, prospective, and open cohort, started in 2013 and including more than 20,000 participants. The i‐Share cohort aims at evaluating multiple health aspects of university students in France, with an annual follow‐up for at least 10 years (Macalli et al., 2020; Montagni et al., 2019). Students were informed about the study through various means, such as social networks, and during university registrations. Eligibility participants had to be (1) formally enrolled at a university or higher education institute in France; (2) at least 18 years of age; and (3) able to read and understand French. Participants answered a baseline self‐questionnaire collecting socio‐demographic, medical and lifestyle information. In Bordeaux, i‐Share students were invited to take a medical examination called Check‐Up, which included an eye examination. Bordeaux students aged between 18 and 35 years were also invited to participate in the MRi‐Share neuroimaging project, which aimed to acquire MRI images in a subsample of the cohort (*N* = 2000) (Mazoyer et al., 2017; Tsuchida, Laurent, Crivello, Petit, Joliot, et al., 2021; Tsuchida, Laurent, Crivello, Petit, Pepe, et al., 2021). All students provided written consent for participation. The MRi‐Share study was approved by the French National Commission of Informatics and Liberties and the study protocol was approved by the local ethics committee (CPP2015‐A00850‐49).

**FIGURE 1:**
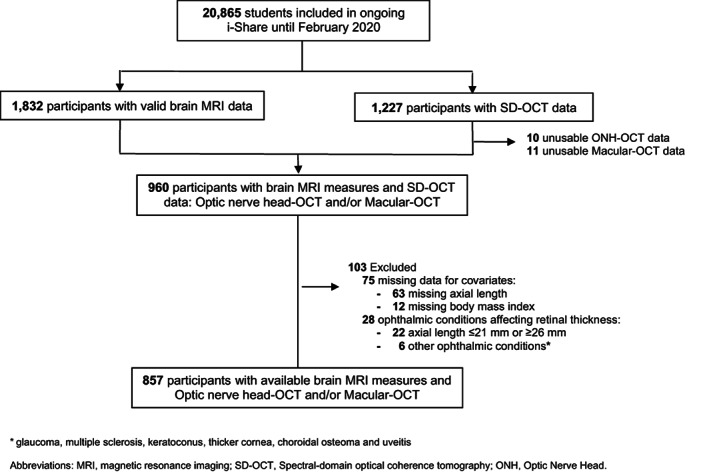
Flow chart of participants, i‐Share study. * glaucoma, multiple sclerosis, keratoconus, thicker cornea, choroidal osteoma and uveitis. MRI, magnetic resonance imaging; ONH, optic nerve head; SD‐OCT, Spectral‐domain optical coherence tomography.

For the present study, we focused on Bordeaux i‐Share participants with cerebral MRI and SD‐OCT data. There were 1832 subjects with valid brain MRI data available from the MRi‐Share project, and 1227 subjects with SD‐OCT data. Of these, 960 individuals had data on both brain and retinal imaging. We excluded 75 participants with missing data for covariates (7.4% for axial length and 1.3% for height and/or weight), and 28 subjects with ophthalmological conditions that could affect retinal nerve thickness layers. Finally, the study sample included 857 participants with available brain MRI data and valid OCT data (857 for cortical morphometry and 854 for diffusion parameters) (Figure 1).

Compared with non‐included eligible participants from the MRi‐Share project (103 subjects from i‐Share Bordeaux with brain MRI and ophthalmic examination), included participants did not differ according to age and sex; however, they were more often past or current smokers (26.4% vs. 17.5%).

### 2.2 MRI acquisition

From November 2015 to November 2017, brain MRI scanning was carried out at the Bordeaux University on the same Siemens Prisma 3‐Tesla with a 64‐channels head coil (gradients: 80 mT/m–200 T/m/s). Exclusion criteria were age over 35 years, any contraindications for MRI, or pregnancy. 3D T1‐weighted and 3D FLAIR, together with 100‐direction 2D diffusion‐weighted imaging (DWI) multiband multi‐shell acquisition were performed. This structural acquisition protocol follows in footsteps that of the UK Biobank brain imaging study (Alfaro‐Almagro et al., 2018) regarding modalities and scanning parameters. Quality control for the brain imaging datasets was performed immediately after the acquisition. Extended description of the MRi‐share protocol has been detailed previously (Tsuchida, Laurent, Crivello, Petit, Joliot, et al., 2021).

### 2.3 MRI processing

Brain images were processed with the Automated Brain Anatomy for Cohort Imaging platform (ABACI, IDDN.FR.001.410013.000.S.P.2016.000.31235) (Tsuchida, Laurent, Crivello, Petit, Joliot, et al., 2021).

The ABACI structural pipeline processed T1 and FLAIR images for multi‐channel volume‐ and surface‐based morphometry. Global image derived phenotypes included total intracranial volume (TIV), total grey matter (GM) and white matter volumes (WM) and mean cortical thickness (CT), all estimated with Freesurfer 6.0 software. The global GM volume includes cortical volume derived from surface‐based analysis and subcortical structures derived from voxel‐based one. The global WM volume is defined as the volume inside the white surface minus anything that is not WM. Cerebral microstructure was examined through DWI data, used to fit (1) diffusion tensor imaging (DTI) modelling and (2) microstructural model fitting with neurite orientation dispersion and density imaging (NODDI). The diffusion parameters reported in this paper are well‐established markers of brain maturation and ageing: fractional anisotropy (FA) and mean diffusivity (MD); and more recent parameters that have been proposed as potential markers for WM microstructural integrity: peak‐width of skeletonized mean diffusivity (PSMD), neurite density index (NDI) and orientation dispersion index (ODI). FA and MD represent respectively the degree of anisotropy of water diffusion, and the amount of diffusivity in all directions in a given voxel. The PSMD, a novel marker for cerebral small vessel disease (Baykara et al., 2016), might be an early marker of ageing because of its increase across the lifespan, in particular from the sixth decade of life (Beaudet et al., 2020). NDI and ODI parameters might provide specific brain microstructure characterization by estimating the neurite density and orientation dispersion with a tissue‐compartment model of diffusion (Zhang et al., 2012). To obtain these global diffusion measurements, all imaging data were processed using a standardized pipeline included in ABACI (see preprocessing details in Tsuchida, Laurent, Crivello, Petit, Joliot, et al. (2021). Preprocessing of DWI data and DTI fitting was performed using tools from the FMRIB software library (FSL) and the dipy package in Python (0.12.0, https://dipy.org) (Garyfallidis et al., 2014), and the AMICO (accelerated microstructure imaging via convex optimization) tool (Daducci et al., 2015) was used for NODDI fitting. A modified script of the PSMD (v0.95, first release, http://www.psmd-marker.com: Baykara et al., 2016) was used to obtain skeletonized DTI and NODDI metrics, based on the FSL tract‐based spatial statistics (TBSS) procedure. The mean FA, MD, NDI, and ODI values were computed from the WM skeleton in the standard MNI space, masked with a skeleton mask provided with the PSMD script. The PSMD was calculated as the difference between the 95th and 5th percentiles of the skeletonized MD values.

Regional GM volume and CT measurements were based on the 34 regions determined by the Desikan‐Killiany atlas segmentation (Desikan et al., 2006). We also analyzed more specifically volumes and CT in three visual areas defined according to the Brodmann's classification: primary visual cortex (V1–BA17), secondary visual cortex (V2–BA18) and associative visual cortex (V5/MT–BA19) (Amunts et al., 2000). Volumes and CT were estimated with FreeSurfer v6.0.

Regional diffusion measures were obtained from the intersection between the 27 ROIs based on the JHU atlas (6 bilateral and 21 left/right WM ROIs) (Mori et al., 2008) and the FSL‐TBSS skeleton output.

As detailed in the publication presenting the acquisition and analysis of the MRi‐Share database (Tsuchida, Laurent, Crivello, Petit, Joliot, et al., 2021), a multi‐stage quality control (QC) process that includes subject‐level qualitative visual QC images as well as quantitative QC metrics ensured the quality of acquired MR images and image derived phenotypes (see Supplementary Material for details), leading to 1832 sets of image derived phenotypes available for the present study.

### 2.4 Eye examination and SD‐OCT measures

Participants underwent an ophthalmic examination, carried out in the Department of Ophthalmology of the Bordeaux University Hospital by a trained health professional, including visual acuity examination, axial length (measured with IntraOcular lens (IOL) Master 500, Zeiss meditec, Dublin, USA) and spectral‐domain OCT scanning.

For each subject, the OCT image acquisition was performed for both eyes without pupil dilatation, using a spectral‐domain OCT scanning (Zeiss Cirrus photo 600, version 1.5.3.23749, Zeiss meditec, Dublin, USA) with images acquisition at a speed of 27,000 A‐scans per second and 5 μm axial resolution in tissue. The optic disc cube acquisition is based on a 3‐dimensional scan of 6 × 6 mm^2^ area centered on the optic disc. A 3.46 mm diameter circular scan was automatically performed around the optic disc that provides 256 measurement points of peripapillary RNFL thickness. Macular thicknesses acquisition was also obtained from a 3‐dimensional scan of 6 × 6 mm^2^ centered on the fovea and the automated segmentation provided by manufacturer software.

Image quality was assessed by trained technicians and scans with signal strength below 7, motion artefacts and segmentation errors were excluded. In this paper, we analyzed the thickness of the peripapillary retinal nerve fiber layer (ppRNFL), macular ganglion cell‐inner plexiform layer (GC‐ILP) and macular ganglion cell complex (GCC) (which includes GC‐IPL and macular RNFL layers). The right eye was primarily selected for analysis in this study. In case of missing values or invalid retinal measurements in the right eye, the left eye was selected. To note, the scale of the retinal layers, measured in μm, is quite different from the scale of structural MRI markers, such as cortical thickness, expressed in mm, or diffusion parameters (RD, AD, MD), nearby 100 μm^2^/s.

### 2.5 Other variables

Demographic and lifestyle factors including sex, age and smoking status, were collected by a self‐questionnaire at baseline. Blood pressure and anthropometric measures were obtained during medical examination. Systolic and diastolic blood pressures were measured twice using a digital monitor.

### 2.6 Statistical analysis

Firstly, we analyzed associations of retinal markers in relation with global brain MRI markers, namely total GM and WM volumes, mean cortical thickness (averaged from the right and left hemispheres), and mean FA, MD, NDI and ODI metrics from WM skeleton. Secondly, we explored associations of retinal markers with GM and WM markers in different brain regions of the Desikan‐Killiany (for GM volume and CT) or JHU (for DTI and NODDI metrics) atlas, without a priori selection, using averaged measures from the right and left hemispheres. Apart from the PSMD, all the diffusion parameters were available for brain region analysis. Multiple linear regression models were used to explore the association of retinal measurements (ppRNFL, GC‐ILP and GCC) with MRI markers (CT, brain volumes, FA, MD, PSMD, NDI and ODI). We calculated z‐scores for each retinal nerve layer thickness by subtracting the mean value from the data value and dividing by the standard deviation (SD). The β coefficients correspond to the variation in neuroimaging outcomes for one SD increase in the retinal nerve layer thickness. For brain volumes or cortical thicknesses, positive β coefficient corresponds to a higher volume or thickness. For diffusion‐based metrics, positive β coefficient corresponds to higher anisotropy for FA, higher overall diffusivity for MD, more variability in the diffusivity for PSMD, higher estimated neurite density for NDI, and more complexity in fiber orientation (or less coherence in the fiber organization) for ODI. While biological interpretations of variability in these metrics in healthy young subjects are not straight‐forward, low FA, high MD, high PSMD, and low NDI are typically observed in aging or pathological conditions and often interpreted as signs of compromised WM microstructure. Thus, the association between a thicker retinal nerve sublayer and a better integrity of the WM microstructure is reflected by positive β coefficient for FA and NDI and negative β coefficient for MD, PSMD and ODI.

The models were adjusted for sex, age, axial length of the eye (which influences the retinal layers thickness), vascular factors (pulse pressure i.e., the averaged difference between the two systolic and diastolic pressures, which had the best Akaike criterion over other blood pressure variables; body mass index, smoking status), and TIV (in order to take into account individual variations in head size). Assumptions of regression models were examined graphically, and the linearity of the quantitative independent variables was verified by multivariable fractional polynomial method. When the linearity was violated, dichotomous variables, based on termplots (<100/≥100 for the ppRNFL, <80/≥80 for the GC‐IPL, <120/≥120 for the GCC) were used. For regional analysis, Benjamini & Hochberg multiple test correction (Benjamini & Hochberg, 1995) for false discovery rate (FDR) was applied separately for each retinal nerve layer. After the FDR correction, a *p*‐value less than or equal to .05 was considered to be statistically significant.

Statistical analyses were performed with R (version 3.6.2; R Core Team).

## 3 RESULTS

### 3.1 Characteristics of the population

The descriptive characteristics of participants are shown in Table 1. The mean (SD) age was 23.3 years (2.6), and 71.3% were female. Regarding vascular risk factors, the mean arterial pulse pressure was 47.8 mmHg (9.9), 2.8% had a high blood pressure (mean blood pressure levels above 140/90 mmHg), the mean (SD) BMI was 22.2 kg/m^2^ (3.6), and more than a quarter of participants were current smokers (26.4%).

**TABLE 1:** Characteristics of the study participants, i‐Share study, *n* = 857.

Characteristics	*N*	Mean ± SD	%
Age, years	857	23.3 ± 2.6	
Sex, %			
Male	246		28.7
Female	611		71.3
Smoking status, %			
No	631		73.6
Current	226		26.4
Pulse pressure, mmHg	857	47.8 ± 9.9	
Systolic blood pressure, mmHg	857	124.6 ± 12.7	
Diastolic blood pressure, mmHg	857	76.7 ± 8.2	
High blood pressure			
No	833		97.2
Yes	24		2.8
Body mass index, kg/m^2^	857	22.2 ± 3.6	
Axial length, mm	857	23.7 ± 0.9	
Peripapillary retinal nerve fiber layer, μm	856	96.5 ± 9.2	
Macular ganglion cell‐inner plexiform layer, μm	857	84.3 ± 6.1	
Macular ganglion cell complex, μm	843	117.4 ± 8.4	
Brain volumes	857		
WM volume, cm^3^		452.9 ± 46.5	
WM fraction, % of TIV		32.6 ± 1.1	
GM volume, cm^3^		676.4 ± 59.4	
GM fraction, % of TIV		48.7 ± 1.6	
TIV, cm^3^		1389.5 ± 125.5	
Cortical thickness, mm	857	2.84 ± 0.08	
Diffusion variables	854		
Fractional anisotropy		0.54 ± 0.01	
Mean diffusivity, 10^−4^ mm^2^/s		7.04 ± 0.16	
Peak‐width of skeletonized mean diffusivity, 10^−4^ mm^2^/s		1.54 ± 0.14	
Neurite density index		0.69 ± 0.027	
Orientation dispersion index		0.20 ± 0.008	

Abbreviations: GM, grey matter; TIV, total intracranial volume; WM, white matter.

The mean retinal nerve layers thicknesses were: 96.5 (9.2) μm for the ppRNFL, 84.3 (6.1) μm for the GC‐IPL and 117.4 (8.4) μm for the GCC. To note, compared with CT (mean value of 2.84 mm), the GCC is about 25 times thinner.

### 3.2 Associations between retinal nerve layers thickness and global MRI parameters

We found few associations between retinal nerve layers and global MRI parameters (Table 2). No associations were found with global GM and WM volumes or mean CT. Concerning diffusion parameters, we found associations between thicker retinal nerve layers and greater FA, significant for GC‐IPL (*p* = .04) and GCC (*p* = .04) and with a trend for ppRNFL (*p* = .06). Thicker retinal nerve layers were also associated with lower PSMD, significantly for ppRNFL (*p* = .009) and with a trend for GC‐IPL (*p* = .05) and GCC (*p* = .06). For NODDI parameters, only one association was found with thicker ppRNFL associated with lower ODI (*p* = .02).

**TABLE 2:** Association between retinal thickness layers and global brain volumes and diffusion markers, i‐Share study, *n* = 857.

	ppRetinal nerve fiber layer			Ganglion cell‐inner plexiform layer			Ganglion cell complex layer	
	β/SD (95% CI)*	*p* value		β/SD (95% CI)*	*p* value		β/SD (95% CI)*	*p* value
Global volumes								
GM volume	0.56 (−2.50; 3.61)	.72		1.13 (−0.37; 2.63)	.14		1.23 (−0.20; 2.67)	.09
WM volume	0.92 (−0.61; 2.44)	.24		0.04 (−1.49; 1.58)	.96		0.34 (−1.05; 1.74)	.63
Cortical thickness (×10^−3^)	−1.39 (−6.88; 4.11)	.62		−3.93 (−9.44; 1.58)	.16		−0.88 (−6.18; 4.42)	.74
Global diffusion markers								
Fractional anisotropy (×10^−4^)	0.96 (−0.03; 1.95)	.06		**1.04 (0.05; 2.04)**	**.04**		**1.02 (0.06; 1.98)**	**.04**
Mean diffusivity (×10^−6^/mm^2^/s)	−0.38 (−1.50; 0.74)	.51		−0.68 (−1.80; 0.44)	.23		−0.57 (−1.65; 0.51)	.30
Peak‐width of skeletonized mean diffusivity (×10^−6^/mm^2^/s)	**−1.23 (−2.15; −0.31)**	**.009**		**−0.93 (−1.8523; 0.0007)**	**.05**		−0.86 (−1.75; 0.03)	.06
Neurite density index (×10^−3^)	0.70 (−1.17; 2.57)	.46		1.01 (−0.86; 2.89)	.29		0.99 (−0.81; 2.79)	.28
Orientation dispersion index (×10^−4^)	**−6.55 (−11.99; −1.12)**	**.02**		−3.57 (−9.02; 1.89)	.20		−4.29 (−9.53; 0.94)	.11

*Note*: Significant associations are highlighted with bold (*p* ≤ .05). For diffusion parameters positive β coefficient for FA and NDI and negative β coefficient for MD, PSMD and ODI correspond to better WM microstructure integrity. Binary ppRNFL for the GM Volume model.

Abbreviations: GM, grey matter; SD, standard deviation; WM, white matter.

* Multiple linear regression models adjusted for sex, age, axial length of the eye, pulse pressure, body mass index, smoking status and total intracranial volume. The β coefficients correspond to the variation in neuroimaging outcomes per one standard deviation increase in the retinal sublayer thickness. For GM and WM a positive β coefficient corresponds to higher volume.

### 3.3 Associations between retinal nerve layers thickness and brain regional volumes and cortical thicknesses

Associations between retinal nerve layers thickness and brain regional volumes are displayed in supplementary material (Figures S1, S2 and S3). After multiple testing corrections, thicker ppRNFL was associated with greater volumes in occipital regions linked to the visual cortex, the pericalcarine (*p* = 10^−9^) and lingual regions (*p* = .0286), and a trend was observed for the cuneus region (*p* = .0645). To note, before the correction, thicker GCC was also associated with greater volume in the pericalcarine region, whereas thicker GC‐IPL was associated with greater volume in the inferior parietal region. No significant associations were found with brain regional cortical thicknesses after multiple testing corrections.

Regarding Brodmann visual areas, after multiple testing corrections, thicker ppRNFL was associated with greater volume in V1‐BA17 (*p* = 10^−7^), while thicker ppRNFL and GC‐IPL were associated with thinner CT in V2–BA18 (*p* = .05 and *p* = .01 respectively) (Table S1).

### 3.4 Associations between retinal nerve layers thickness and regional white matter diffusion parameters

**FIGURE 2:**
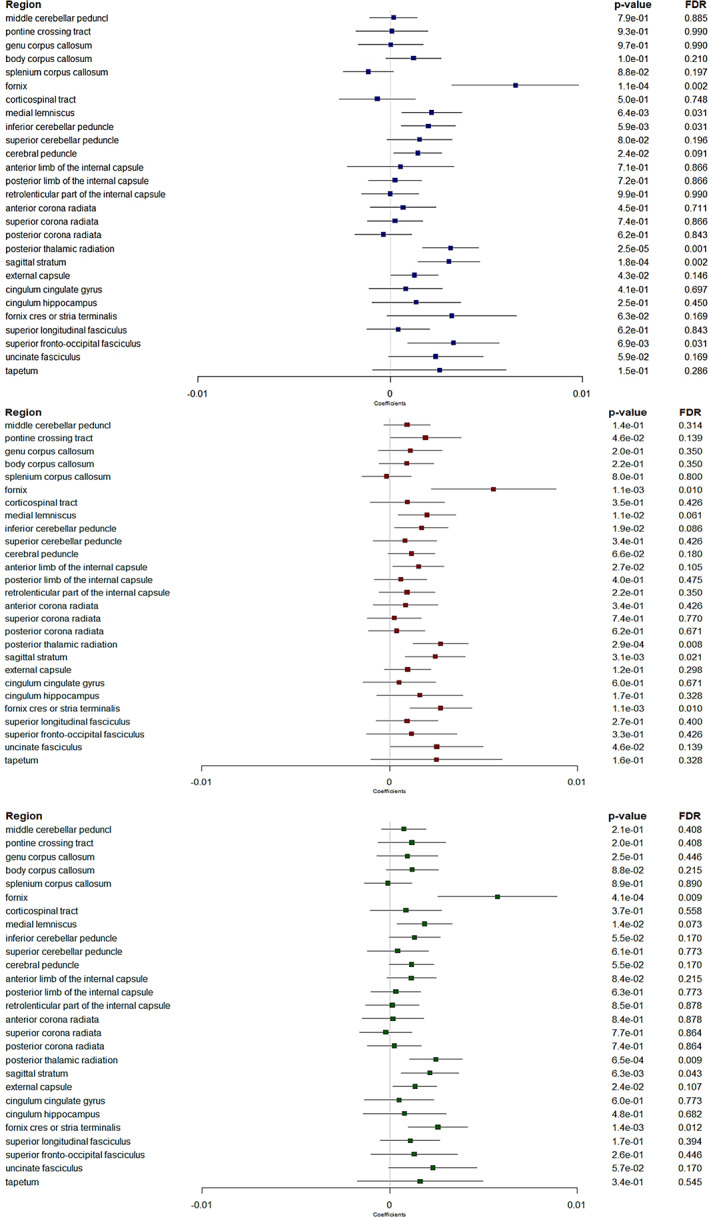
Forest plots showing associations between retinal layers and fractional anisotropy. Boxes represent coefficients and horizontal lines represent 95% Confidence Intervals (non‐corrected). peripapillary RNFL (blue). GC‐IPL (red). GCC (green). Multiple linear regression models adjusted for sex, age, axial length of the eye, pulse pressure, body mass index, smoking status and total intracranial volume. A positive β coefficient corresponds to an increase in FA better WM microstructure integrity, per one standard deviation increase in the retinal sublayer thickness. FDR, false discovery rate. Dichotomous ppRNFL for the fornix cres or stria terminalis region.

**FIGURE 3:**
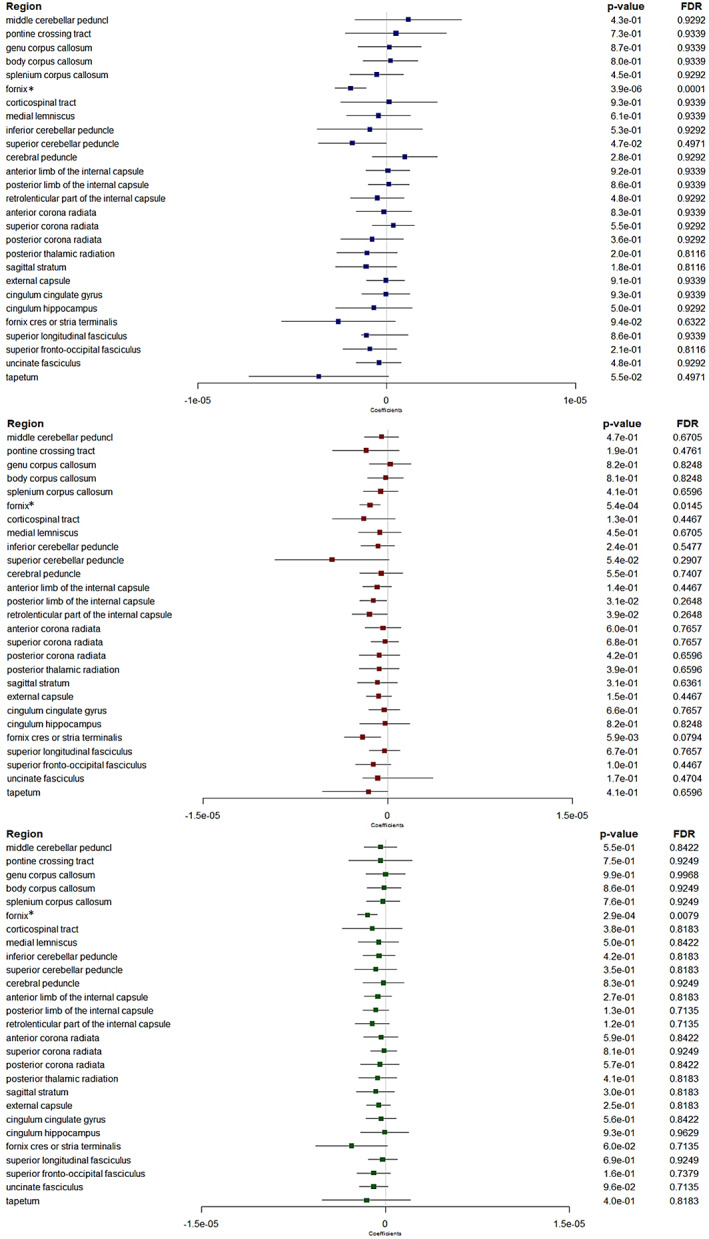
Forest plots showing associations between retinal layers and mean diffusivity. Boxes represent coefficients and horizontal lines represent 95% Confidence Intervals (non‐corrected). peripapillary RNFL (blue). GC‐IPL (red). GCC (green). Multiple linear regression models adjusted for sex, age, axial length of the eye, pulse pressure, body mass index, smoking status and total intracranial volume. A negative β coefficient corresponds to a decrease in mean diffusivity better WM microstructure integrity, per one standard deviation increase in the retinal sublayer thickness. FDR, false discovery rate. *Log‐transformed outcomes: change in the scale of the coefficients and confidence intervals for presentation reasons. Dichotomous retinal layers in the regions: ppRNFL for the middle cerebellar peduncle, inf cerebellar peduncle; ppRNFL and GCC for the fornix cres or stria terminalis.

**FIGURE 4:**
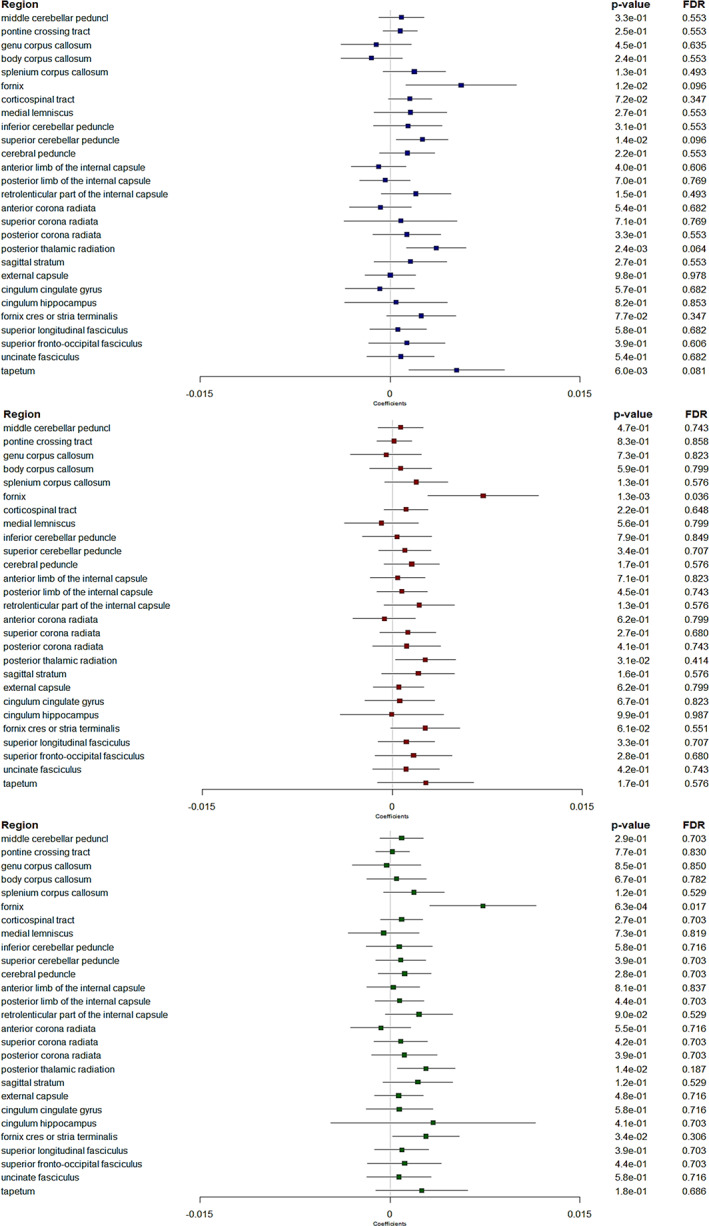
Forest plots showing associations between retinal layers and neurite density index. Boxes represent coefficients and horizontal lines represent 95% Confidence Intervals (non‐corrected). peripapillary RNFL (blue). GC‐IPL (red). GCC (green). Multiple linear regression models adjusted for sex, age, axial length of the eye, pulse pressure, body mass index, smoking status and total intracranial volume. A positive β coefficient corresponds to an increase in neurite density index better WM microstructure integrity, per one standard deviation increase in the retinal sublayer thickness. FDR, false discovery rate. Dichotomous retinal layers in the regions: ppRNFL for the sup corona radiata, and GCC for the cingulum hippocampus.

**FIGURE 5:**
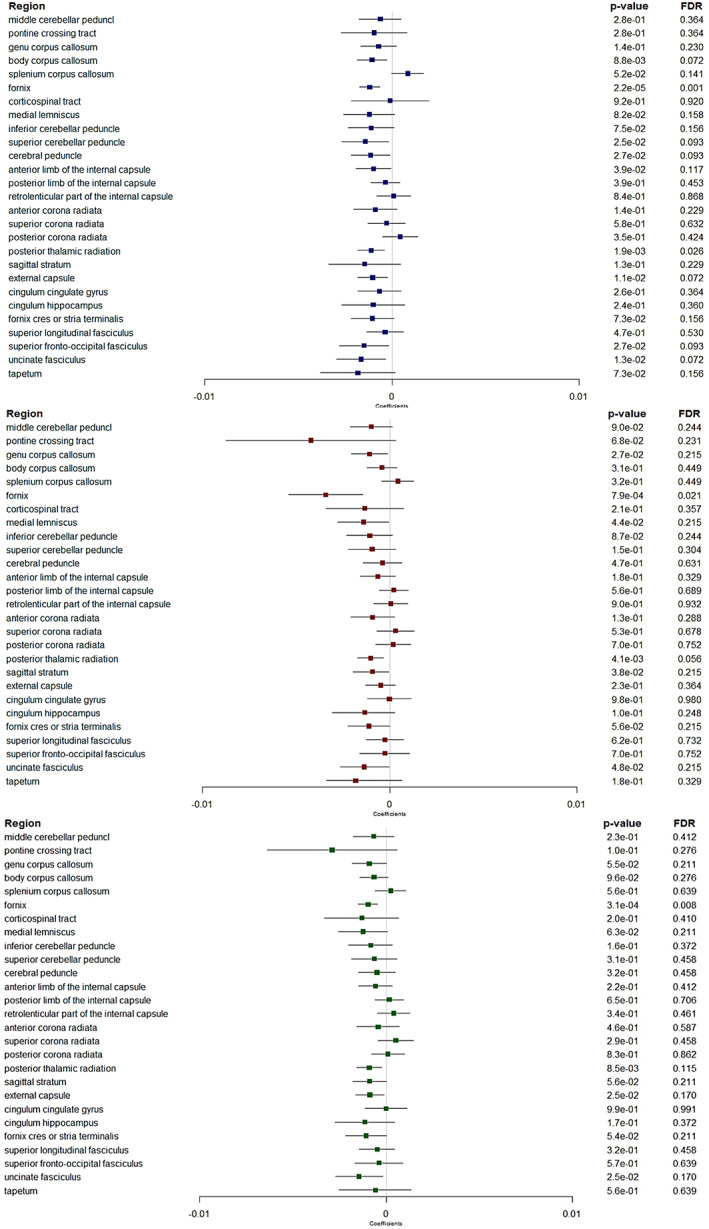
Forest plots showing associations between retinal layers and orientation dispersion index. Boxes represent coefficients and horizontal lines represent 95% Confidence Intervals (non‐corrected). peripapillary RNFL (blue). GC‐IPL (red). GCC (green). Multiple linear regression models adjusted for sex, age, axial length of the eye, pulse pressure, body mass index, smoking status and total intracranial volume. A negative β coefficient corresponds to a decrease in orientation dispersion index better WM microstructure integrity, per one standard deviation increase in the retinal sublayer thickness. FDR, false discovery rate. Dichotomous retinal layers in the regions: GC‐IPL and GCC for the pontine crossing tract.

Non‐corrected analyses showed several significant associations, with thicker retinal nerve layers associated with patterns of diffusion metrics consistent with higher WM microstructural integrity in several regions (Figures 2, 3, 4, 5). Many of these associations remained significant after correction for multiple testing, in particular in a region involved in the visual pathway and in association regions.

*FA and MD markers* (Figures 2 and 3). Globally, associations were more marked for FA than for MD. After correction for multiple testing, thicker ppRNFL, GC‐IPL and GCC were associated with higher FA in the post thalamic radiation region (*p* = .001, *p* = .008 and *p* = .009, respectively), which includes the optic radiations and is linked to the visual cortex. Besides, thicker ppRNFL, GC‐IPL and GCC were associated with higher FA in the sagittal stratum (*p* = .002, *p* = .021 and *p* = .043, respectively), a major cortico‐subcortical WM bundle. Higher GC‐IPL and GCC thicknesses were associated with higher FA in another association region, the fornix cres or stria terminalis (*p* = .010 and *p* = .012, respectively).

In addition, thicker ppRNFL, GC‐IPL and GCC were associated with higher FA (*p* = .002, *p* = .010 and *p* = .009, respectively), and lower MD (*p* = .0001, *p* = .0145 and *p* = .0079, respectively) in the fornix. And thicker ppRNFL was also associated with higher FA in the superior fronto‐occipital fasciculus, and in the brainstem regions (medial lemniscus and inferior cerebellar peduncle) (*p* = .031 for each of the three).

*NDI and ODI markers* (Figures 4 and 5). After correction for multiple testing, thicker ppRNFL was associated with lower ODI in the post thalamic radiation region (*p* = .026). Although not significant, there were also trends for thicker ppRNFL with higher NDI (*p* = .064) and thicker GC‐IPL with lower ODI (*p* = .056) in the same region. Similar pattern was also observed in the fornix, with thicker ppRNFL, GC‐IPL and GCC associated with lower ODI (*p* = .001, *p* = .021 and *p* = .008 respectively) and thicker GC‐IPL and GCC with higher NDI in the fornix too (*p* = .036 and *p* = .017).

## 4 DISCUSSION

This study is, to the best of our knowledge, the first that explored the retina‐brain links in a large sample of healthy young individuals. In this young population, retinal‐brain associations were found mainly with diffusion parameters allowing detecting more subtle changes in the integrity of the WM microstructure. As expected, we found associations with several brain regions involved in the visual pathway. In this pathway, not only diffusion parameters, but also GM volumes were associated with retinal layers, likely reflecting more direct and strong associations. However, our results have shown associations beyond visual regions, mainly in regions containing association fiber bundles, suggesting that retinal layers may reflect brain WM microstructure more globally, and not limited to the fibers of the visual pathway. This is reinforced by the associations we founded between retinal layer thickness and global diffusion parameters. Altogether, these findings support our hypothesis that retinal layer might reflect brain microstructure in young people.

Most of the previous studies on the associations between retinal layers and brain MRI markers analyzed global or regional tissue volumes rather than the microstructure of the WM. In non‐diseased populations, they found that thinner retinal layers was associated with atrophy in the brain (Barrett‐Young et al., 2023; Casaletto et al., 2017; Chua et al., 2021; Jorge et al., 2020; Mauschitz et al., 2022; Mejia‐Vergara et al., 2021; Méndez‐Gómez et al., 2018; Mutlu et al., 2017, 2018; Ong et al., 2015; Shi et al., 2020). However, most of them were carried out in elderly or middle‐aged populations with only two including young subjects under 30 years old, but no more than 20 individuals, preventing any analysis specifically in the young population (Jorge et al., 2020; Mejia‐Vergara et al., 2021). Among these studies, one found that thicker RNFL was associated with greater volume in an occipital region linked to the visual cortex, more precisely the pericalcarine region (Mejia‐Vergara et al., 2021), as in our study, albeit in a much small sample of 20 subjects between age of 27 and 71 years. Consistently, when analyzing more specific visual areas, we also found an association between thicker ppRNFL and greater volume in the primary visual cortex (V1‐BA17). Moreover, thicker ppRNFL and GC‐IPL were also associated with thinner CT in the secondary visual cortex (V2–BA18).

Previous investigations conducted specifically in older populations reported associations between retinal thinning and reduced GM and WM brain volumes globally (Mutlu et al., 2017; Ong et al., 2015) and in different areas including: (i) the occipital lobe or visual areas (Méndez‐Gómez et al., 2018; Mutlu et al., 2017, 2018; Ong et al., 2015; Shi et al., 2020); and (ii) the temporal or mediotemporal lobe or the hippocampus, which are regions associated with Alzheimer's disease. (Casaletto et al., 2017; Méndez‐Gómez et al., 2018; Mutlu et al., 2017; Ong et al., 2015). Most of these studies have focused on the thicknesses of two retinal layers, the ppRNFL and the macular ganglion cell layer.

Findings from recent studies including middle‐aged participants are quite similar to those of studies including only older adults, but some of them additionally examined other retinal layers than the ppRNFL and the ganglion cell layer (Chua et al., 2021; Mauschitz et al., 2022). In the UK Biobank (Chua et al., 2021) (*n* = 2131) and in the Rhineland Study (*n* = 2872) (Mauschitz et al., 2022), most of retinal layers were associated with brain volumes including overall volumes and GM volumes in the occipital pole and the hippocampus (Chua et al., 2021). In the Dunedin Multidisciplinary Health and Development Study (*n* = 825), thinner ppRNFL and GC‐IPL were associated with reduced subcortical volumes, but also with reduced cortical surface and thinner average cortex (Barrett‐Young et al., 2023). However, contrary to most previous studies, in their parcel‐wise analyses, the associations found with retinal layers were widely distributed across the cortex, rather than regionally‐specific.

In our young population, we did not find significant associations with global structural MRI markers (i.e., GM/WM volumes or cortical thickness), but only with diffusion parameters reflecting WM microstructure. Apparent discrepancies with prior studies may be due to differences in the age of the included populations; indeed, the associations in middle‐aged and older adults could reflect age‐related neurodegenerative processes.

Beyond the analysis of brain morphometric measures, very few studies have examined WM microstructural properties in relation to retinal layers. In our study, thicker retinal nerve layers were associated with higher WM microstructural integrity patterns (higher FA, lower PSMD, and, to a lesser extent, a combination of higher NDI and lower ODI) globally and in several areas including: (i) the post thalamic radiation region, which is involved in the visual pathway; (ii) the sagittal stratum (and occasionally in adjacent external capsule) region composed of projection and association fibers including the inferior longitudinal fasciculus (IFO) and the inferior longitudinal fasciculus (ILF), that are neighbor tracts of the optic radiation (Mori et al., 2008); (iii) and the fornix, another association bundle belonging to the limbic system. It is interesting that the pattern of association with DTI and NODDI metrics was consistent across these regions, and consistent with the interpretation that subjects with thicker retinal layers have diffusion properties associated with higher WM integrity: higher FA and lower PSMD (indicating less variability in the amount of diffusivity along the core WM skeleton) suggest more coherence of WM fibers overall. Although biological interpretations of DTI metrics can be unspecific (e.g., FA is sensitive to both the underlying fiber composition of a given voxel and microstructural integrity, such as myelination and axon density), higher FA coupled with higher NDI and lower ODI suggests that thicker retinal layers are associated with both higher axon density and/or myelination and less dispersion of fiber orientations, that is, higher fiber coherence, in subjects with thicker retinal layers.

It is also interesting that aforementioned regions with significant associations between retinal layers and FA, NDI and/or ODI metrics are WM regions that are part of visual or limbic memory pathways with strong functional associations with cortical and subcortical regions implicated in previous studies in older subjects. These regional patterns and the direction of changes in DTI metrics are also consistent with few studies that investigated the relationships between the retinal layers and WM properties.

In the Rotterdam cohort, reduced ppRNFL and reduced ganglion cell layer were associated with altered WM microstructure (lower FA and higher MD) globally (Mutlu et al., 2017) and in WM tracts in the visual pathway using a voxel‐based analysis (Mutlu et al., 2018). In the Three‐City Study‐Bordeaux cohort, thicker ppRNFL was associated with preserved cerebral microstructure (higher FA and lower MD) in regions including the visual pathway and those involved in neurodegenerative process of Alzheimer's disease (Méndez‐Gómez et al., 2018). Moreover, in the Rhineland Study including middle‐aged participants, thicker inner retinal layers mainly (including ppRNFL, ganglion cell layer, and inner plexiform layer) were associated with preserved WM microstructure (globally and in the optic radiation area) (Mauschitz et al., 2022).

We had hypothesized that retinal macular layers, which include cell bodies of retinal neurons (i.e., GC‐IPL and GCC), should reflect more the condition of the cerebral GM, while the ppRNFL, which is composed of axons, may reflect more the cerebral WM condition. This assumption was based on the physiological composition of the retinal layers and on one previous study showing that thinning of the GC‐IPL was associated with GM, but not WM volume (Ong et al., 2015). Our results were not concordant with this assumption as we have shown associations on one hand, between ppRNFL and GM volume in occipital areas, and on the other hand, between all retinal layers with WM microstructure. Several previous studies assessing peripapillary and macular layers also found that both layers were associated with GM and WM (Barrett‐Young et al., 2023; Mauschitz et al., 2022; Mutlu et al., 2017, 2018).

Given the specific age range of our population, the maturational changes in both the microstructural architecture of brain WM and the retinal sublayers make the study of the relationship between the two very complex and likely could impact our results. Knowledge about maturational changes at both retinal and brain levels is still incomplete. While the retinal layers become thinner with aging (Cheung et al., 2011; Duan et al., 2010; Hoffmann et al., 2018; Mauschitz et al., 2018; Nousome et al., 2021; von Hanno et al., 2017), studies including children, adolescents or young adults rather suggest a thickening of retinal layers up to young adults ages, although studies are scarce and only cross‐sectional (Cheng et al., 2019; Huynh et al., 2008; Mwanza et al., 2011). Regarding the brain, previous large cross‐sectional and longitudinal brain MRI studies focusing on structural and diffusion tensor markers and including young adults have reported common patterns between the 20's and 40's, that is, WM volume increase, cortical thinning, increases in FA and decreases in MD (Coupé et al., 2017; Kochunov et al., 2011; Koolschijn & Crone, 2013; Lebel et al., 2012; Lebel & Beaulieu, 2011; Pines et al., 2020; Simmonds et al., 2014; Tamnes et al., 2010; Westlye et al., 2010; Yeatman et al., 2014; Zhou et al., 2015). Results from the MRi‐Share cohort included in the present study also reported the same patterns in subsample of subjects aged between 18 and 26 (Tsuchida, Laurent, Crivello, Petit, Joliot, et al., 2021). A recent multi‐cohort study with a large collective sample (*n* = 20,005) across the adult lifespan confirmed these patterns of age‐related DTI changes (Beaudet et al., 2020). Age‐related changes in NODDI metrics are less well‐understood, but we have previously shown that among the students of the MRi‐Share study NDI increase globally with age, while age‐related variations in ODI are more regionally circumscribed (Tsuchida, Laurent, Crivello, Petit, Joliot, et al., 2021; Tsuchida, Laurent, Crivello, Petit, Pepe, et al., 2021). These strong maturational changes in both FA, NDI, and retinal layers (all increasing) in young adults concord with the association found between thicker retinal nerve layers and greater FA and NDI. The lowest change with age of ODI could in part explain our observation of a thicker retinal nerve layer associated with lower ODI.

The retinal‐brain connection revealed in our study posits questions about its implications: can the retinal layers also be related to variations in cognitive performance? Some studies conducted in young people suggested that brain MRI changes may be related to cognitive performance in different domains (Botdorf et al., 2022; Estrada et al., 2019; Lee et al., 2014; Tamnes et al., 2010, 2013). However, their cross‐sectional design or short‐term follow‐up preclude drawing conclusions about longer‐term cognition. On the other hand, studies exploring the associations between retinal layers and cognitive function in young populations are very scarce. Indeed, the previous studies that have explored these associations were mainly conducted among adults (over 40 years) (Girbardt et al., 2021; Jeevakumar et al., 2022; Khawaja et al., 2016; Ko et al., 2018; Merten et al., 2020). To our knowledge, only one previous study in this field has included young people, showing that retinal layer thickness assessed in middle age was associated with cognitive performance in childhood and adulthood (Barrett‐Young et al., 2022). Although retinal layer thickness was not assessed in childhood in this study, the results are in favor of a link between retinal layers and cognition even early in the life.

A major strength of our study is its large sample size, with retinal data and brain imaging data collected independently, using standardized protocols. Besides, to the best of our knowledge, this is the first large study assessing at the same time retinal layers and brain MRI markers in a sample composed exclusively of young healthy subjects. Moreover, we could have access to novel diffusion‐based measures that might allow a detailed characterization of the white matter microstructural architecture in our population. Finally, we explored the associations between retinal layers and numerous brain regions, without a priori selection, taking into account multiple testing. This work has also some limitations. First, given the cross‐sectional design of our study, it was not possible to examine temporal associations between retina and brain; however, this study aimed to investigate the parallel maturation processes of retinal and brain structures, with the assumption that retinal structures may mirror brain structures. Nevertheless, longitudinal data are necessary to further understand the evolution of the retina and brain over the time. Second, our sample is composed of young university students, predominantly female, thus it may not be representative of population of this age‐range in general. Third, we excluded participants with missing data on covariates; however, given the influence of axial length on retinal layer thickness and the low proportion of missing data in our sample, we decided not to apply imputation. Fourth, the associations have not been consistently found with the different brain parameters analyzed in our study.

## 5 CONCLUSION

The retina is anatomically an extension of the brain. In this young healthy population, for which the cerebral maturation is still strongly in progress, we have found relationships between some retinal nerve layers and diffusion MRI parameters in regions containing associative tracts and in a projection area involved in the visual pathway. Beyond the anatomical origin, these results suggest common maturational processes in the retina and the brain. Further studies are needed to confirm these results in young subjects, and to document whether common factors may explain the parallel maturation of the retina and the brain. Moreover, additional studies should also investigate more in‐depth the associations within the visual cortex using high resolution MRI as well as the links with cognitive function, to understand whether retinal‐brain associations have functional repercussions.

## FUNDING INFORMATION

The i‐Share team is currently supported by an unrestricted grant of the Nouvelle‐Aquitaine Regional Council (Conseil Régional Nouvelle‐Aquitaine, grant N°4370420). It has also received grants from the Nouvelle‐Aquitaine Regional Health Agency (Agence Régionale de Santé Nouvelle‐Aquitaine, grant N°6066R‐8), Public Health France (Santé Publique France, grant N°19DPPP023‐0), and The National Institute against cancer INCa (Institut National Du Cancer, grant N°INCa_11502). The funding bodies were neither involved in the study design, or in the collection, analysis, or interpretation of the data. Digital Public Health Graduate Program, with funding support from the framework of the PIA3 (Investment for the future). Project reference 17‐EURE‐0019.

## CONFLICT OF INTEREST STATEMENT

Sara Cristina Lima Rebouças, Fabrice Crivello, Ami Tsuchida, Christophe Tzourio and Catherine Helmer have no conflicts of interest to disclose. Cédric Schweitzer is consultatnt for Abbvie, Alcon, Glaukos, Nicox, Théa, Horus, Johnson & Johnson, Santen and Bausch & Lomb. Jean‐François Korobelnik is consultant for Abbvie, Apellis, Bayer, Janssen, Nanoretina, Roche, Théa and Carl Zeiss Meditec. Cecile Delcourt is consultant for Allergan, Chauvin‐Bausch+Lomb, Laboratoires Théa and Novartis.

## INFORMED CONSENT STATEMENT

All participants signed a written informed consent form.

## Supporting information


**Data S1:** Supporting Information.

## Data Availability

The datasets generated and analyzed during the current study are not publicly available, as they contain information that could compromise research participant consent (i‐Share & MRi‐Share). However, data are available from the corresponding author upon reasonable request and with permission of the principal investigators of the cohorts.
