# Supplementary material for: Association of retinal nerve layers thickness and brain imaging in healthy young subjects from the i‐Share‐Bordeaux study
Source: Hum Brain Mapp. 2023 Jul 4;44(13):4722–37. doi: 10.1002/hbm.26412 (PMC10400793; doi:10.1002/hbm.26412)
Supplement: Supplementary file 1 — Data S1: Supporting Information. [file HBM-44-4722-s001.docx]

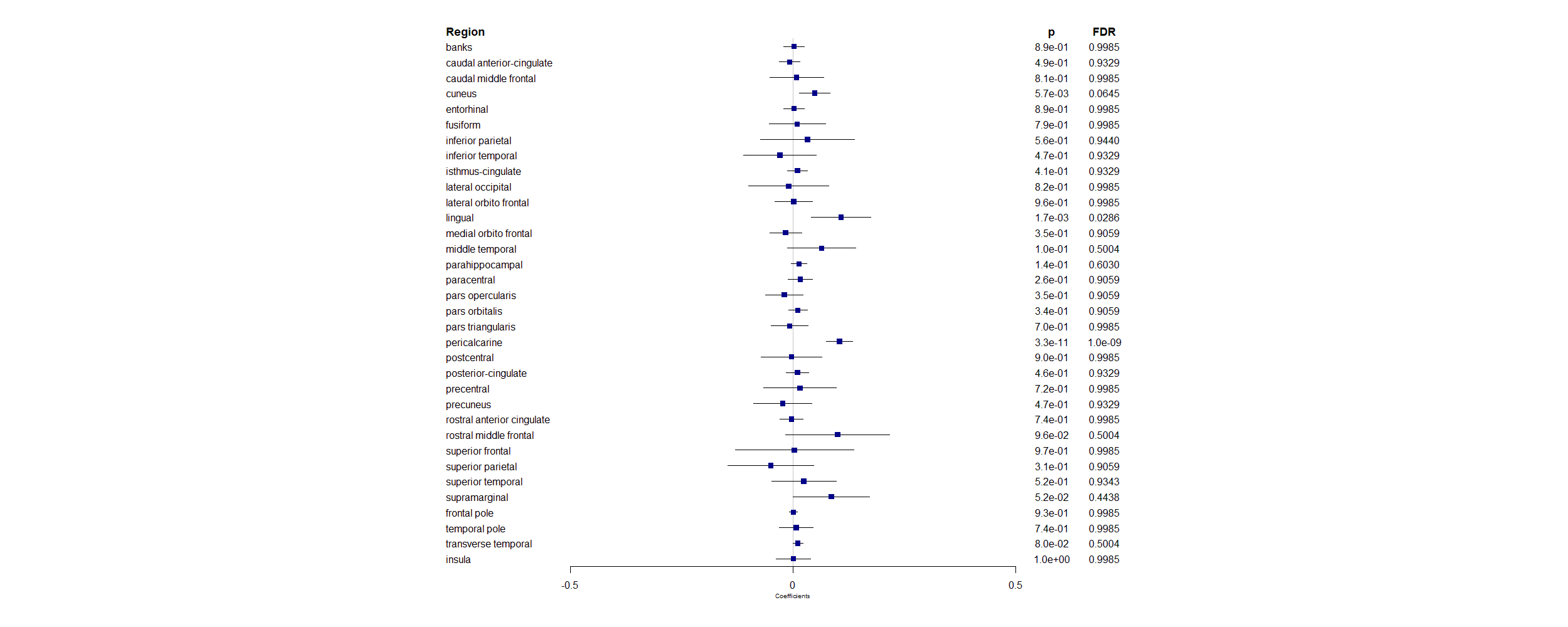
Supplementary material

Abbreviations: FDR, false discovery rate.

Supplementary Figure 1. Forest plot showing associations between peripapillary RNFL and brain regional volumes.

Multiple linear regression models adjusted for sex, age, axial length of the eye, pulse pressure, body mass index, smoking status and total intracranial volume. Boxes represent coefficients and horizontal lines represent 95% Confidence Intervals (non-corrected). A positive β coefficient corresponds to an increase in both peripapillary RNFL and brain volumes. Binary RNFL for the temporal pole model.


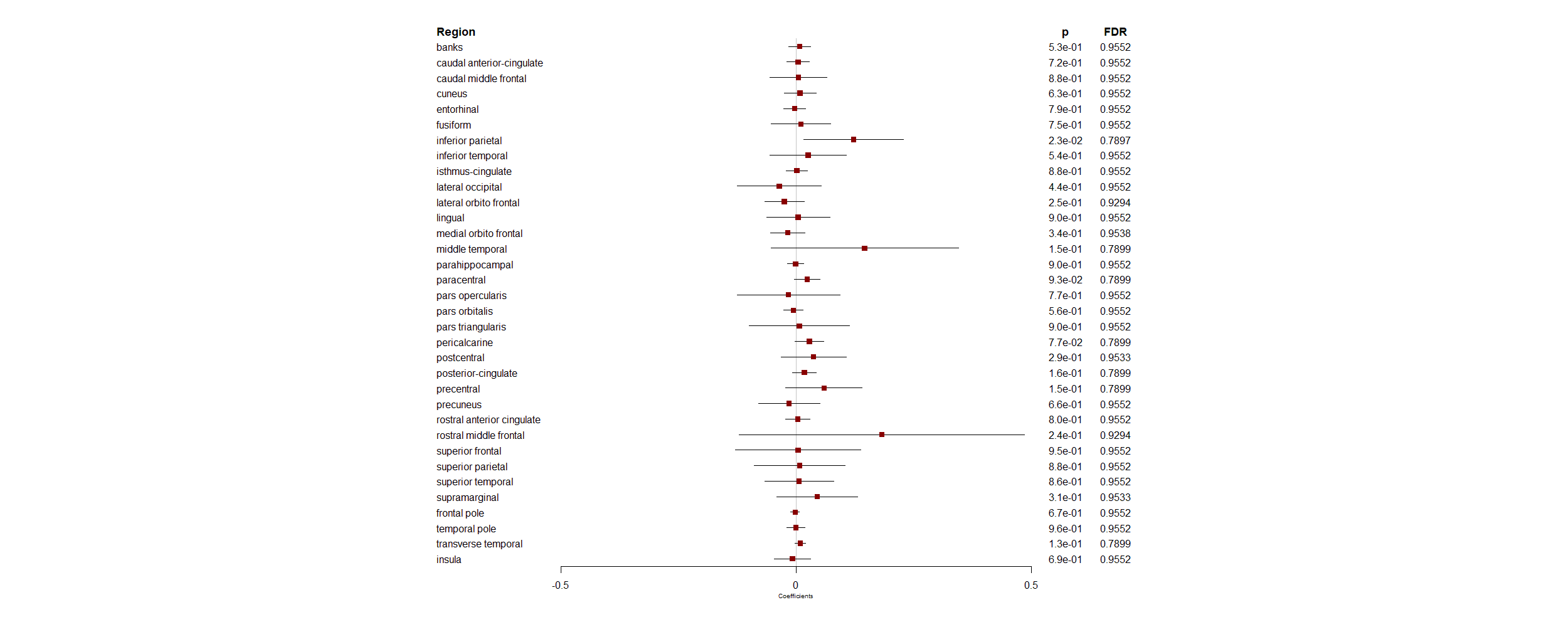


Abbreviations: FDR, false discovery rate.

Supplementary Figure 2. Forest plot showing associations between GC-IPL and brain regional volumes.

Multiple linear regression models adjusted for sex, age, axial length of the eye, pulse pressure, body mass index, smoking status and total intracranial volume. Boxes represent coefficients and horizontal lines represent 95% Confidence Intervals (non-corrected). A positive β coefficient corresponds to an increase in both GC-IPL and brain volumes. Binary GC-IPL for the middle temporal, pars opercularis, pars triangularis and rostral middle frontal models.


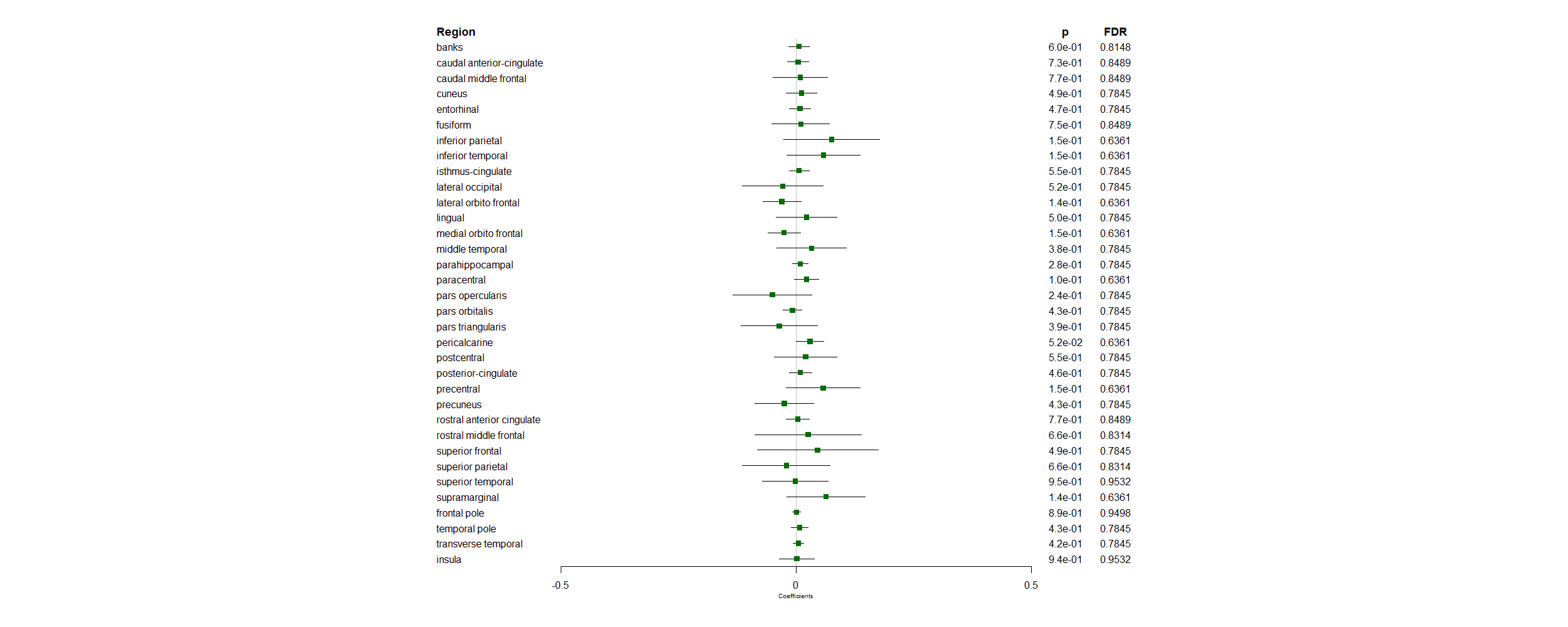


Abbreviations: FDR, false discovery rate.

Supplementary Figure 3. Forest plot showing associations between GCC and brain regional volumes.

Multiple linear regression models adjusted for sex, age, axial length of the eye, pulse pressure, body mass index, smoking status and total intracranial volume. Boxes represent coefficients and horizontal lines represent 95% Confidence Intervals (non-corrected). A positive β coefficient corresponds to an increase in both GCC and brain volumes. Binary GCC for the pars opercularis and pars triangularis models.

Supplementary Table 1. Association between retinal thickness layers and regional volumes or regional cortical thicknes in areas linked to the visual system (Brodmann classification), i-Share study, n = 857.

|  | | ppRetinal nerve fiber layer | | | | |  | | Ganglion cell-inner plexiform layer | | | | | |  | | Ganglion cell complex layer | | | | | |  |
| --- | --- | --- | --- | --- | --- | --- | --- | --- | --- | --- | --- | --- | --- | --- | --- | --- | --- | --- | --- | --- | --- | --- | --- |
|  | | β (95% CI)* | | p value | | FDR |  | | β (95% CI)* | | p value | | FDR | |  | | β (95% CI)* | | p value | | FDR | |  |
| Brodmann areas - volumes | |  | |  | |  |  | |  | |  | |  | |  | |  | |  | |  | |  |
| Primary visual cortex (V1) | | 0.13 (0.08 ; 0.18) | | 6.5^e-08^ | | 2.0^e-07^ |  | | 0.02 (-0.03 ; 0.07) | | 0.46 | | 0.46 | |  | | 0.03 (-0.02 ; 0.07) | | 0.27 | | 0.61 | |  |
| Secondary visual cortex (V2) | | 0.07 (-0.02 ; 0.17) | | 0.13 | | 0.20 |  | | -0.04 (-0.14 ; 0.06) | | 0.41 | | 0.46 | |  | | -0.02 (-0.11 ; 0.07) | | 0.68 | | 0.70 | |  |
| Associative visual cortex (V5) | | 0.01 (-0.03 ; 0.04) | | 0.58 | | 0.58 |  | | 0.02 (-0.01 ; 0.06) | | 0.26 | | 0.46 | |  | | 0.01 (-0.02 ; 0.05) | | 0.40 | | 0.61 | |  |
| Brodmann areas – cortical thickness |  | |  | |  | | |  | |  | |  | |  | |  | |  | |  | |  | |
| Primary visual cortex (V1) | 0.0004 (-0.0078 ; 0.0086) | | 0.93 | | 0.93 | | |  | | -0.0081 (-0.0163 ; 0.0001) | | 0.05 | | 0.08 | |  | | -0.0075 (-0.0154 ; 0.0004) | | 0.06 | | 0.09 | |
| Secondary visual cortex (V2) | -0.009 (-0.017 ; -0.002) | | 0.02 | | 0.05 | | |  | | -0.011 (-0.019 ; -0.004) | | 0.004 | | 0.01 | |  | | -0.009 (-0.016 ; -0.001) | | 0.02 | | 0.06 | |
| Associative visual cortex (V5) | 0.001 (-0.007 ; 0.009) | | 0.82 | | 0.93 | | |  | | -0.003 (-0.011 ; 0.005) | | 0.45 | | 0.45 | |  | | -0.001 (-0.009 ; 0.006) | | 0.75 | | 0.75 | |

* Multiple linear regression models adjusted for sex, age, axial length of the eye, pulse pressure, body mass index, smoking status and total intracranial volume. The β coefficients correspond to the variation in neuroimaging outcomes per one standard deviation increase in the retinal sublayer thickness. A positive β coefficient corresponds to higher volume or thinner cortical thickness.

**Supplemental Methods.** **MRI Processing**

The quality control steps for MRI processing were extensively described previously (Tsuchida et al., 2021). Different procedures were used to structural and diffusion MRI processing quality control. For the structural MRI processing, the images were reviewed by three experienced and qualified MD investigators for any incidental and non-incidental findings and for visible artefacts, including ringing and reduced contrast in the raw images. Then, a trained rater evaluated the flagged images on four categories according to the rating system proposed by Backhausen *et al*. (2016) (Backhausen et al., 2016); 1) Image sharpness, 2) Ringing, 3) Contrast to noise ratio (CNR) of subcortical structures, and 4) CNR of GM and WM. A score was assigned for each category, given such that 0, 1, and 2 represented ‘good’, ‘moderate’, and ’bad’ quality as described by Backhausen *et al*. (2016) (Backhausen et al., 2016). Besides, another trained rater (N.B) inspected individual qualitative QC images, independently of the initial flagging of the artefacts, originated by the pipeline used for all scanned participants. This last rater also inspected another set of images and movies using a modified version of ENIGMA Cortical QC scripts (package 2.0, April 2017: http://enigma.ini.usc.edu/protocols/imaging-protocols/), which generated multiple views of Freesurfer surface reconstructions and cortical and subcortical parcellations to detect any gross failures in surface reconstruction. No major failures were found at the global surface reconstructions among the 1,832 subjects with valid MRI data, during the inspection; however, segmentation problems were identified at the regional level. Finally, there no extreme outliers were detected in global surface-based measures including mean cortical thickness or in total grey and with matter volumes. Regarding the diffusion MRI processing quality control, both qualitative and quantitative quality control metrics were computed for each subject. These metrics were generated based on different tools including FSL EDDY (Bastiani et al., 2019) and other in-house custom tools inspired by QC described in Tournier *et al*. (2011) (Tournier et al., 2011) and Roalf *et al*. (2016) (Roalf et al., 2016)”

References

Backhausen, L. L., Herting, M. M., Buse, J., Roessner, V., Smolka, M. N., & Vetter, N. C. (2016). Quality Control of Structural MRI Images Applied Using FreeSurfer—A Hands-On Workflow to Rate Motion Artifacts. *Frontiers in Neuroscience*, *10*. https://doi.org/10.3389/fnins.2016.00558

Bastiani, M., Cottaar, M., Fitzgibbon, S. P., Suri, S., Alfaro-Almagro, F., Sotiropoulos, S. N., Jbabdi, S., & Andersson, J. L. R. (2019). Automated quality control for within and between studies diffusion MRI data using a non-parametric framework for movement and distortion correction. *NeuroImage*, *184*, 801–812. https://doi.org/10.1016/j.neuroimage.2018.09.073

Roalf, D. R., Quarmley, M., Elliott, M. A., Satterthwaite, T. D., Vandekar, S. N., Ruparel, K., Gennatas, E. D., Calkins, M. E., Moore, T. M., Hopson, R., Prabhakaran, K., Jackson, C. T., Verma, R., Hakonarson, H., Gur, R. C., & Gur, R. E. (2016). The impact of quality assurance assessment on diffusion tensor imaging outcomes in a large-scale population-based cohort. *NeuroImage*, *125*, 903–919. https://doi.org/10.1016/j.neuroimage.2015.10.068

Tournier, J.-D., Mori, S., & Leemans, A. (2011). Diffusion tensor imaging and beyond: Diffusion Tensor Imaging and Beyond. *Magnetic Resonance in Medicine*, *65*(6), 1532–1556. https://doi.org/10.1002/mrm.22924

Tsuchida, A., Laurent, A., Crivello, F., Petit, L., Joliot, M., Pepe, A., Beguedou, N., Gueye, M.-F., Verrecchia, V., Nozais, V., Zago, L., Mellet, E., Debette, S., Tzourio, C., & Mazoyer, B. (2021). The MRi-Share database: Brain imaging in a cross-sectional cohort of 1870 university students. *Brain Structure and Function*, *226*(7), 2057–2085. https://doi.org/10.1007/s00429-021-02334-4
